# Burden and trends of brain and central nervous system cancer from 1990 to 2019 at the global, regional, and country levels

**DOI:** 10.1186/s13690-022-00965-5

**Published:** 2022-09-17

**Authors:** Yang Fan, Xian Zhang, Chao Gao, Shuai Jiang, Haoze Wu, Zhanhui Liu, Taotao Dou

**Affiliations:** Department of Neurosurgery, Ninth Hospital of Xi’an, Xi’an, 710054 China

**Keywords:** Incidence, Mortality, Disability-adjusted life years, Central nervous system cancer, Brain cancer

## Abstract

**Background:**

Regularly updated epidemiological data on the burden of brain and central nervous system (CNS) cancers are important in the prioritization of research and the allocation of resources. This study aimed to investigate incidence, mortality, disability, and trends in brain and CNS cancers between 1990 and 2019.

**Methods:**

Epidemiological data, including the cancer incidence, mortality, disability-adjusted life years(DALYs), age-standardized incidence rate (ASIR), age-standardized mortality rate (ASMR), and age-standardized DALY rate (per 100,000 population) stratified by region, country, sex, and age group were retrieved and extracted using the Global Health Data Exchange (GHDx) query tool.

**Results:**

In 2019, there were 347,992(262,084–388,896) global cases of brain and CNS cancers, which showed a significant increase (94.35%) from the period between 1990 to 2019. The global ASIR in 2019 was 4.34 (3.27–4.86) per 100, 000 population, which showed an increasing trend for the years 1990–2019 (13.82% [-27.27–32.83]). In 2019, there were 246,253 (185,642–270,930) global deaths caused by brain and CNS cancers, which showed a significant increase (76.36%) during the study period. The global ASMR in 2019 was 3.05(2.29–3.36) per 100, 000 population, which did not change significantly over the study period (-1.19% [-36.79–13.86]). In 2019, there were 8,659,871 DALYs, which was a 109.04% increase compared with 1990. Similarly, during 1990–2019, the age-standardized DALY rate decreased by 10.39%. Additionally, 76.60% of the incident cases, 72.98% of the deaths, and 65.16% of the DALYs due to brain and CNS cancers occurred in the high-income and upper-middle-income regions.

**Conclusions:**

In conclusion, brain and CNS cancers remain a major public health burden, particularly in high-income regions. The global incidence, deaths, and DALYs of brain and CNS cancers were shown to have increased significantly from 1990 to 2019. The global ASIR kept rising steadily, while the ASMR and age-standardized DALY rate declined over the past three decades.

**Supplementary Information:**

The online version contains supplementary material available at 10.1186/s13690-022-00965-5.

## Background

Brain and central nervous system (CNS) cancers include a complex and varied group of malignant cancers deriving from the brain and its surrounding structures. According to Global Cancer Statistics 2020, 308,102 new cases of brain and CNS cancers were diagnosed, and 251,329 cancer-related deaths occurred in 2020 worldwide [1]. Previously, brain and CNS cancers have been found to be in the top ten causes of death and the second leading cause of cancer deaths in adolescents [2, 3]. Moreover, research has identified brain and CNS cancers as the second greatest contributors to the disability-adjusted life-year (DALY) burden among adolescent cancer [4]. Brain and CNS cancers can affect children, adults, and the elderly, and can occur in any area of the CNS. They have been shown to be the main cause of morbidity worldwide, causing a high burden on healthcare systems along with their high mortality rate, high disability, and reduced quality of life [5]. With regard to the causes of brain and CNS cancers, studies have found that brain and CNS cancers may be due to individual genetic variations, environmental factors, or occupational exposure [6–9]. The diagnosis and treatment of brain and CNS cancers demand highly specialized and multidisciplinary medical care, which varies greatly from region to region and lead to the discrepancies in clinical outcomes. In general, the clinical outcomes were not entirely suboptimal in low-income and middle-income regions [10].The socioeconomic status of a country may affect the burden of brain and CNS cancers. Some studies have observed positive associations between the occurrence of brain and CNS cancers and socioeconomic characteristics [11–13]. However, systemic epidemiological data on the burden of brain and CNS cancers in countries with different income levels is somewhat lacking. The incidence of brain and CNS cancers can vary according to histological subtype, country, race, age, and sex. With the improvement of the scanning and diagnostic techniques and changes in environmental risk factors, the incidence of brain and CNS cancers is expected to increase. Additionally, the extended lifespan coupled with the higher tumors detection rates have caused higher incidence of elderly cancers. Updated epidemiological data on the burden of brain and CNS cancers is thus required. However, there are few reports on the epidemiology of brain and CNS cancers in all age groups [14–16]. Some studies have focused only on the incidence and mortality of brain and CNS tumors in children, while ignoring incidence and mortality in elderly patients. Moreover, the latest epidemiological data are not updated in a timely manner. Information on the epidemiology of brain and CNS cancers is important for clinical practice and research in neuro-oncology. This will be important in the prioritization of research and the allocation of resources for cancer surveillance and primary prevention. Therefore, this study presents an up-to-date summary of incidence, mortality, disability, and trends for brain and CNS cancers in all age groups at the global, regional, and country levels, based on data from the 2019 Global Burden of Disease (GBD) Study.

## Methods

### Data sources

Data sources for the disease burden of brain and CNS tumors were collected using the Global Health Data Exchange (GHDx) query tool (http://ghdx.healthdata.org/gbd-results-tool). These data were summarized in the 2019 GBD Study [17]. Epidemiological data, including the cancer incidence, mortality, DALYs, age-standardized incidence rate (ASIR), age-standardized mortality rate (ASMR), and age-standardized DALY rate (per 100,000 population) stratified by region, country, sex, and age group were retrieved and extracted. The methods to calculate estimations of these metrics were the same as the previous literature [18].The 204 countries and territories in the GBD study were divided into five regions based on the sociodemographic index (SDI) or 21 GBD regions based on geography. The SDI is an index ranging from 0.0 (lowest) to 1.0 (highest), and is a composite indicator of income per capita, average educational attainment, and total fertility rates [19]. In 2019 GBD Study, brain and CNS cancer includes all cancers coded as C70.0–C72.9 (C70, malignant neoplasm of meninges; C71, malignant neoplasm of brain; C72, malignant neoplasm of spinal cord, cranial nerves, and other parts of the CNS) in the International Classification of Diseases tenth revision.

### Statistical analysis

The ASIR, ASMR, and age-standardized DALY rates were used to compare the differences across the different regions/countries or time periods. These metrics were calculated by the formula $ASR=\sum_{i=1}^{A}{a}_{i}{w}_{i}/\sum_{i=1}^{A}{w}_{i}\times 100,000$, where ${a}_{i}$ represents the age-specific rates, and ${w}_{i}$ represents the number of person in the same age group *i*, and *A* represents the number of age groups in the reference standard population [20]. Smooth curve fitting was used to explore the relationship between the SDI of different countries and age-standardized rates of brain and CNS cancers in these countries [21]. A joinpoint regression analysis was conducted to determine the trends in the burden of brain and CNS cancers between 1990–2019. We used Joinpoint software (version 4.7.0) to perform this analysis and to obtain the annual percent changes (APCs) in the three age-standardized rates [22]. All the statistical analyses were performed using R software (version 4.0.5). The statistical significance was set at *P* < *0.05*.

## Results

### Global burden of brain and CNS cancers

In 2019, 347,992 (95% uncertainty interval [UI] 262,084–388,896) global cases of brain and CNS cancers were reported, which was a significant increase (94.35% [95% UI 21.95–128.32]) for the years 1990 to 2019 (Tables 1 and 2). The global ASIR in 2019 was 4.34 (3.27–4.86) per 100, 000 population, which indicated an increasing, though not significant, trend for the years 1990–2019 (13.82% [-27.27–32.83]). In the same year, there were 246,253(185,642–270,930) global deaths that were caused by brain and CNS cancers, which was a significant increase (76.36% [11.02–104.86]). The global ASMR in 2019 was 3.05 (2.29–3.36) per 100, 000 population, which did not change significantly from 1990 to 2019 (-1.19% [-36.79–13.86]). In 2019, there were 8,659,871 (6,718,029–9,574,458) DALYs, which was a 109.04% (84.57–120.92) increase from 1990. Similarly, during 1990–2019, the age-standardized DALY rate decreased by 10.39%, which was not significant (-43.49–5.26). In 2019, the regions with middle SDI levels had the highest incidence, death, and DALYs for brain and CNS cancers (98,924 [73,328–113,227] incident cases, 73,757 [53,504–83,949] deaths, and 2,663,892 [1,965,774–3,015,123] DALYs) (Table 1). The incident cases, deaths, and DALYs occurred most frequently in the high, high-middle, and middle SDI regions (Table 1). Moreover, 76.60% of incident cases, 72.98% of deaths, and 65.16% of DALYs due to brain and CNS cancers occurred in the high-income and upper-middle-income regions in 2019 (Table 1).

**Table 1:** The numbers and age-standardized rates of brain and central nervous system cancer in global in 2019

**Variables**	**Incidence numbers (95% UI)**	**Age-standardized incidence rate per 100, 000 (95% UI)**	**Death numbers (95% UI)**	**Age-standardized mortality rate per 100, 000 (95% UI)**	**DALYs numbers (95% UI)**	**Age-standardized DALY rate per 100, 000 (95% UI)**
Global	347,992 (262,084–388,896)	4.34 (3.27–4.86)	246,253 (185,642–270,930)	3.05 (2.29–3.36)	8,659,871 (6,718,029–9,574,458)	109.04 (84.57–120.92)
Sex						
Female	160,501 (113,748–184,350)	3.89 (2.78–4.46)	107,648 (76,392–121,708)	2.56 (1.82–2.88)	3,646,543 (2,697,490–4,102,434)	90.85 (67.80–102.01)
Male	187,491 (135,414–215,390)	4.84 (3.49–5.56)	138,605 (99,626–157,036)	3.58 (2.55–4.05)	5,013,328 (3,660,068–5,743,657)	127.56 (92.92–146.21)
Regions categorized by SDI						
High SDI quintile	91,689 (63,988–105,543)	6.46 (4.63–7.43)	54,835 (38,310–59,957)	3.45 (2.50–3.74)	1,545,128 (1,148,349–1,671,947)	118.21 (89.35–127.86)
High-middle SDI quintile	97,785 (70,828–110,647)	5.64 (4.12–6.37)	68,769 (49,107–77,088)	3.69 (2.65–4.12)	2,186,778 (1,635,277–2,428,430)	131.60 (97.82–146.54)
Middle SDI quintile	98,924 (73,328–113,227)	4.01 (2.95–4.59)	73,757 (53,504–83,949)	2.99 (2.16–3.39)	2,663,892 (1,965,774–3,015,123)	107.69 (79.39–121.96)
Low-middle SDI quintile	41,624 (32,890–48,780)	2.63 (2.08–3.07)	34,471 (27,280–40,051)	2.26 (1.78–2.62)	1,489,754 (1,187,633–1,743,318)	89.28 (71.02–104.15)
Low SDI quintile	17,813 (13,105–21,958)	2.01 (1.47–2.42)	14,305 (10,592–17,500)	1.80 (1.34–2.15)	769,950 (570,156–957,835)	73.08 (53.96–89.66)
Regions categorized by the World Bank income level						
World Bank High Income	111,535 (76,618–130,160)	6.57 (4.64–7.64)	68,666 (46,684–75,598)	3.61 (2.54–3.94)	1,920,873 (1,379,901–2,092,226)	123.18 (91.54–133.37)
World Bank Upper Middle Income	155,017 (113,594–177,148)	5.16 (3.80–5.89)	111,052 (81,122–126,434)	3.51 (2.54–4.00)	3,722,154 (2,788,165–4,206,049)	126.94 (94.80–142.88)
World Bank Lower Middle Income	70,927 (54,295–81,867)	2.51 (1.91–2.88)	57,911 (44,298– 66,117)	2.13 (1.63–2.44)	2,560,328 (1,954,236–2,955,164)	85.13 (64.69–98.20)
World Bank Low Income	10,355 (7,502–13,035)	1.94 (1.39–2.42)	8,507 (6,102–10,620)	1.77 (1.26–2.18)	452,133 (330,335–578,738)	69.99 (50.40–87.78)

*Abbreviations*: *UI* Uncertainty interval, *DALY* Disability-adjusted life-year, *SDI* Socio-demographic index

**Table 2:** Percentage change of numbers and age standardized rates of brain and central nervous system cancer for 1990–2019

**Variables**	**% change in incidence numbers**	**% change in age-standardized incidence rate**	**% change in death numbers**	**% change in age-standardized mortality rate**	%change in DALYs numbers	**%change in age-standardized DALY rate**
Global	94.35 (21.95–128.32)	13.82 (-27.27–32.83)	76.36 (11.02–104.86)	-1.19 (-36.79–13.86)	40.46 (-13.18–66.89)	-10.39 (-43.49–5.26)
Sex						
Female	102.59 (13.64–154.07)	17.40 (-32.42–46.28)	77.64 (1.14–123.01)	-1.71 (-42.84–22.53)	41.39 (-22.63–80.00)	-10.77 (-49.85–12.69)
Male	87.82 (21.19–131.32)	11.10 (-27.25–34.70)	75.37 (17.32–112.55)	-0.87 (-32.97–18.62)	39.80 (-7.74–78.71)	-10.01 (-39.52–12.07)
Regions categorized by SDI						
High SDI	84.24 (13.14–115.87)	19.70 (-25.02–39.39)	60.95 (-1.51–78.23)	-2.59 (-38.84–6.91)	35.26 (-12.88–47.45)	-8.36 (-40.60–0.51)
High-middle SDI	90.17 (25.14–119.69)	22.35 (-20.27–40.89)	63.41 (5.81–87.66)	-3.07 (-37.77–11.21)	27.82 (-15.98–45.87)	-13.31 (-43.76–0.66)
Middle SDI	99.01 (21.55–137.46)	15.63 (-27.86–37.78)	92.05 (20.25–128.55)	3.59 (-33.66–23.10)	41.06 (-15.10–71.99)	-9.00 (-44.32–9.63)
Low-middle SDI	82.13 (7.68–139.62)	12.89 (-29.15–40.92)	87.63 (14.89–140.63)	10.38 (-28.26–35.44)	45.63 (-16.68–103.01)	-0.32 (-39.58–30.26)
Low SDI	98.33 (17.24–191.25)	5.34 (-34.02–41.06)	115.00 (27.71–212.16)	12.59 (-29.50–49.75)	93.17 (10.42–204.26)	4.34 (-36.77–48.70)
Regions categorized by the World Bank income level						
World Bank High Income	77.00 (7.57–108.51)	17.60 (-27.04–38.44)	55.60 (-6.19–72.94)	-3.66 (-40.50–5.99)	29.52 (-18.07–41.37)	-10.36 (-42.91– -2.55)
World Bank Upper Middle Income	107.58 (30.93–147.95)	27.89 (-19.49–52.60)	79.68 (14.32–113.44)	0.56 (-36.44–19.50)	30.87 (-19.07–57.08)	-11.96 (-45.76–5.41)
World Bank Lower Middle Income	95.52 (20.34–150.24)	12.79 (-25.39–37.72)	95.37 (23.60–143.83)	8.45 (-26.64–29.86)	59.75 (-6.04–114.28)	0.72 (-36.73–27.18)
World Bank Low Income	106.32 (19.32–217.52)	9.27 (-32.51–52.38)	112.42 (24.34–218.23)	11.37 (-30.36–51.92)	93.87 (7.71–217.53)	3.64 (-38.39–51.58)

*Abbreviations*: *DALY* Disability-adjusted life-year, *SDI* Socio-demographic index

### Regional and national burden of brain and CNS cancers

**Fig. 1:**
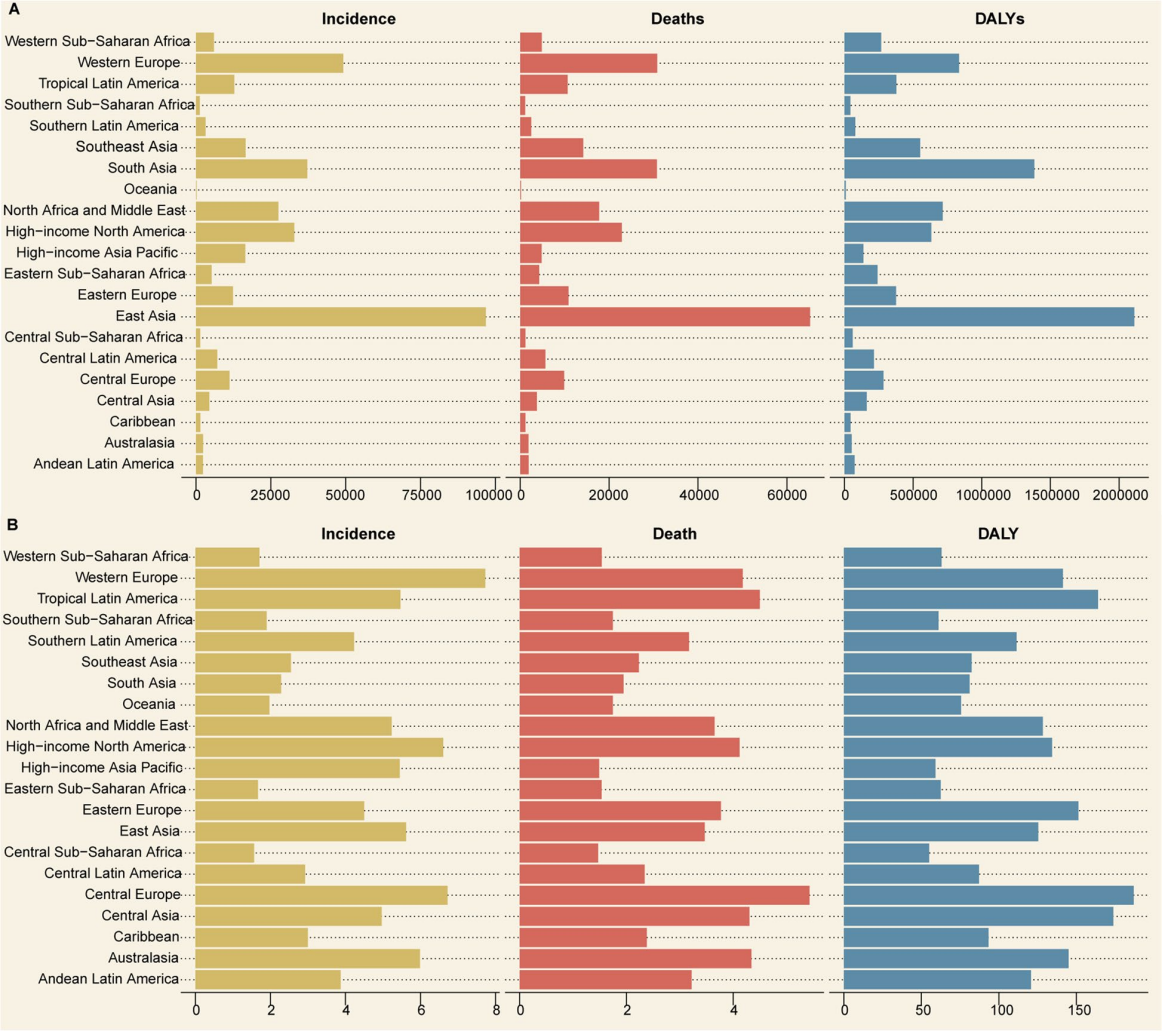
The numbers (**A**) and age-standardized rates (**B**) of brain and central nervous system cancer in 21 GBD regions in 2019. Abbreviations: GBD, Global Burden of Disease

**Fig. 2:**
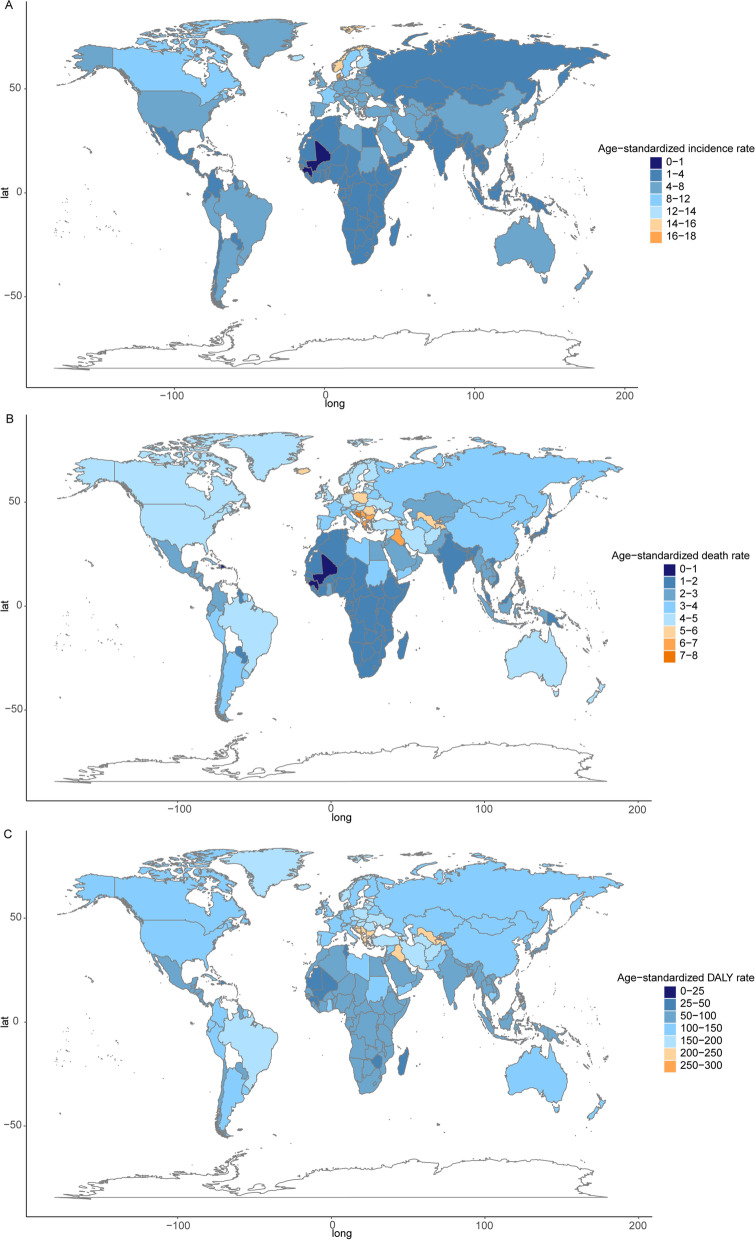
Global map of age-standardized rate (per 100,000 populations) of brain and central nervous system cancer in 2019, by country and territory. **A** age-standardized incidence rate; **B** age-standardized mortality rate; **C** age-standardized DALY rate. Abbreviations: DALY, disability-adjusted life-year

At the regional level, among the 21 GBD regions in 2019, incident cases, deaths, and DALYs occurred most frequently in East Asia, Western Europe, and South Asia (Fig. 1A, Table 3). Furthermore, at the regional level, the ASIRs were highest in Western Europe, Central Europe, and high-income North America (Fig. 1B, Table 3). The ASMRs were the highest in Central Europe, tropical Latin America, and Australasia (Fig. 1B, Table 3), while the age-standardized DALY rates were highest in Central Europe, Central Asia, and tropical Latin America (Fig. 1B, Table 3). At the national level, the highest ASIR was observed in San Marino (17.45 [12.56–23.74] per 100, 000 population), Denmark (17.12 [10.11–23.02] per 100, 000 population), Andorra (16.78 [11.97–22.34] per 100, 000 population), Norway (15.53 [10.73–18.67] per 100, 000 population), and Iceland (13.94 [10.20–16.91] per 100, 000 population; Fig. 2A) among the 204 countries and territories. The highest ASMRs were observed in San Marino (7.32 [4.66–10.99] per 100, 000 population), Palestine (7.24 [5.24–8.68] per 100, 000 population), Montenegro (7.19 [5.47–9.04] per 100, 000 population), Bosnia and Herzegovina (7.02 [3.99–9.21] per 100, 000 population), and Serbia (6.85 [4.68–8.91] per 100, 000 population; Fig. 2B).The highest age-standardized DALY rates were observed in Montenegro (253.47 [194.43–325.59] per 100, 000 population), San Marino (250.43 [160.63–375.78] per 100, 000 population), Bulgaria (235.80 [138.83–321.11] per 100, 000 population), Andorra (234.68 [167.69–314.54] per 100, 000 population), and Palestine (232.00 [175.63–279.46] per 100, 000 population; Fig. 2C).

**Table 3:** The numbers and age-standardized rates of brain and central nervous system cancer in 21 GBD regions in 2019

**GBD region**	**Incidence numbers (95% UI)**	**Age-standardized incidence rate per 100, 000 (95% UI)**	**Death numbers (95% UI)**	**Age-standardized mortality rate per 100, 000 (95% UI)**	**DALYs numbers (95% UI)**	**Age-standardized DALY rate per 100, 000 (95% UI)**
Andean Latin America	2,327 (1,327–3,090)	3.87 (2.20–5.15)	1,895 (1,075–2,519)	3.22 (1.82–4.28)	74,544 (42,675–99,388)	120.73 (69.12–160.67)
Australasia	2,359 (1,679–2,969)	5.99 (4.31–7.55)	1,858 (1,347–2,082)	4.34 (3.20–4.83)	52,959 (39,522–58,641)	144.95 (109.80–161.15)
Caribbean	1,477 (718–1,980)	3.00 (1.47–4.04)	1,187 (575–1,574)	2.38 (1.16–3.20)	44,544 (23,221–61,984)	93.33 (48.89–131.94)
Central Asia	4,450 (3,028–5,334)	4.96 (3.39–5.92)	3,752 (2,551–4,496)	4.30 (2.95–5.12)	162,291 (110,000–194,835)	173.86 (118.70–208.26)
Central Europe	11,199 (7,500–13,395)	6.72 (4.69–7.96)	9,900 (6,502–11,914)	5.43 (3.71–6.48)	284,711 (199,650–338,657)	187.00 (135.02–221.26)
Central Latin America	7,129 (4,876–8,780)	2.92 (1.98–3.61)	5,656 (3,830–6,883)	2.34 (1.58–2.85)	214,934 (146,733–262,470)	87.18 (59.41–106.64)
Central Sub-Saharan Africa	1,408 (1,015–1,827)	1.56 (1.09–2.00)	1,192 (850–1,533)	1.47 (1.01–1.89)	60,581 (43,922–83,050)	55.01 (38.97–70.56)
East Asia	96,818 (75,148–116,192)	5.62 (4.31–6.68)	65,213 (48,948–78,580)	3.47 (2.61–4.15)	2,110,764 (1,624,281–2,585,838)	125.41 (95.89–152.69)
Eastern Europe	12,392 (9,524–14,300)	4.50 (3.53–5.15)	10,878 (8,267–12,609)	3.77 (2.92–4.34)	376,535 (295,284–432,917)	151.27 (119.24–173.02)
Eastern Sub-Saharan Africa	5,238 (4,093–6,686)	1.67 (1.27–2.06)	4,261 (3,304–5,383)	1.54 (1.15–1.86)	241,252 (188,469–315,817)	62.46 (48.19–78.61)
High-income Asia Pacific	16,476 (9,039–20,240)	5.45 (3.17–6.49)	4,813 (2,651–5,598)	1.49 (0.86–1.69)	138,622 (79,209–158,998)	59.17 (36.25–66.66)
High-income North America	32,807 (24,835–38,210)	6.60 (5.13–7.72)	22,861 (17,512–24,540)	4.12 (3.23–4.44)	632,594 (507,341–691,962)	134.31 (109.47–148.48)
North Africa and Middle East	27,529 (18,554–32,579)	5.23 (3.50–6.15)	17,773 (12,096–20,936)	3.65 (2.45–4.30)	716,271 (493,932–848,226)	128.34 (87.81–151.30)
Oceania	225 (143–353)	1.97 (1.30–2.90)	183 (118–280)	1.75 (1.17–2.51)	9,951 (6,084–16,584)	75.63 (48.24–116.75)
South Asia	37,191 (28,419–44,752)	2.28 (1.73–2.74)	30,748 (23,853–36,824)	1.95 (1.51–2.34)	1,383,155 (1,081,667–1,662,443)	81.08 (63.40–97.76)
Southeast Asia	16,553 (10,921–19,911)	2.55 (1.68–3.06)	14,172 (9,388–17,247)	2.23 (1.48–2.70)	552,008 (379,459–669,067)	82.44 (57.08–99.53)
Southern Latin America	3,181 (2,371–4,056)	4.23 (3.18–5.39)	2,469 (1,975–2,717)	3.17 (2.55–3.48)	79,742 (66,146–88,353)	111.42 (93.28–123.57)
Southern Sub-Saharan Africa	1,258 (874–1,443)	1.90 (1.32–2.18)	1,107 (767–1,263)	1.75 (1.19–2.00)	43,759 (31,002–50,646)	61.04 (42.81–69.96)
Tropical Latin America	12,789 (8,350–14,620)	5.47 (3.54–6.28)	10,669 (6,725–12,189)	4.50 (2.81–5.15)	377,708 (253,179–429,298)	163.96 (109.09–187.27)
Western Europe	49,192 (31,359–60,930)	7.73 (5.05–9.52)	30,828 (19,197–34,682)	4.18 (2.68–4.65)	834,861 (544,320–926,527)	141.33 (93.22–155.87)
Western Sub-Saharan Africa	5,994 (3,703–8,301)	1.71 (0.99–2.26)	4,839 (3,009–6,750)	1.54 (0.90–2.08)	268,084 (171,305–388,450)	63.04 (39.24–87.40)

*Abbreviations*: *GBD* Global Burden of Disease, *DALY* Disability-adjusted life-year, *UI* Uncertainty interval

### Trends in the ASIRs, ASMRs, and age-standardized DALY rates from 1990 to 2019

**Fig. 3:**
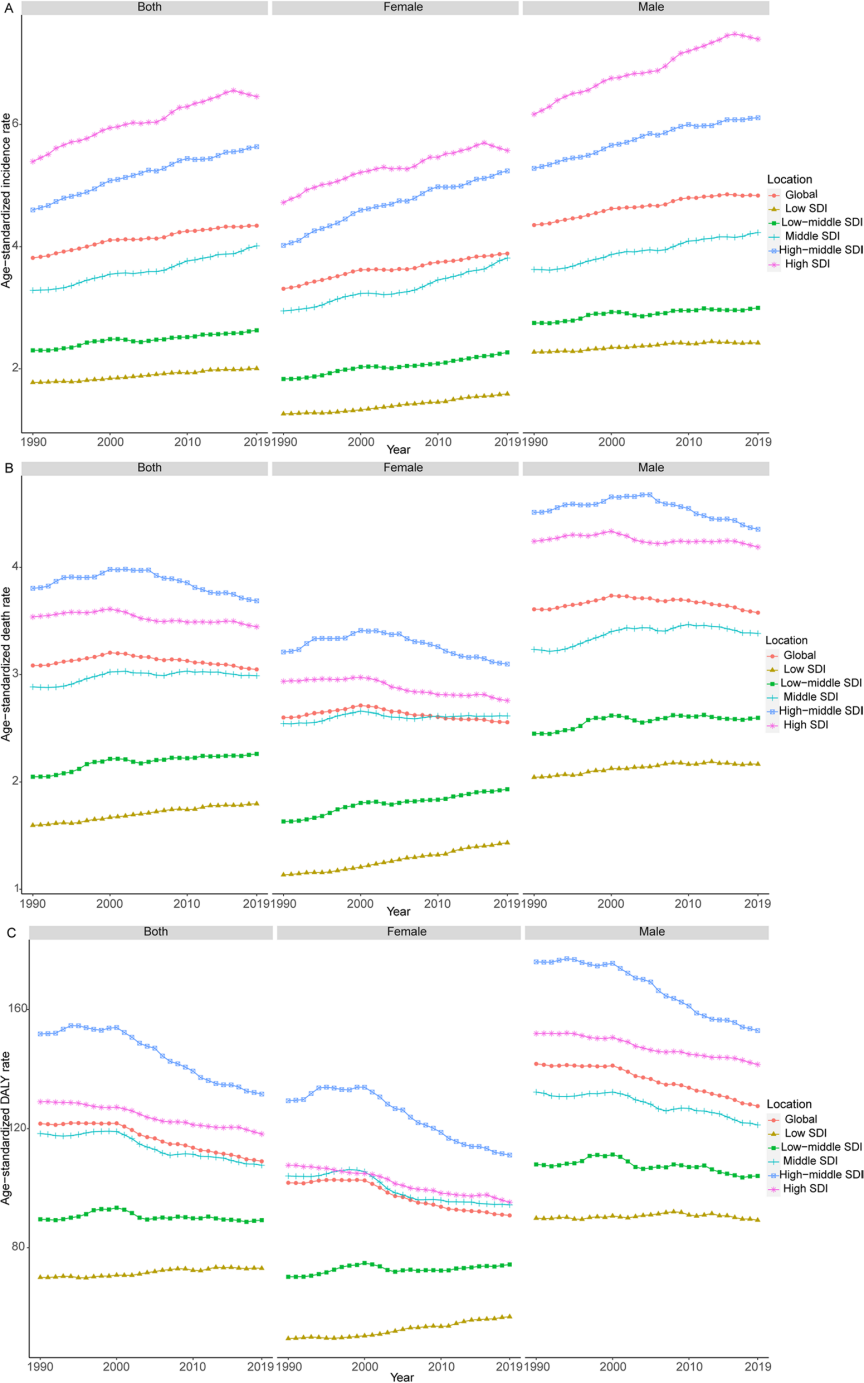
The ASIR (**A**), ASMR (**B**), and age-standardized DALY rate (**C**) of brain and central nervous system cancer from 1990 to 2019. Abbreviations: ASIR, age-standardized incidence rate; ASMR, age-standardized mortality rate; DALY, disability-adjusted life-year

**Fig. 4:**
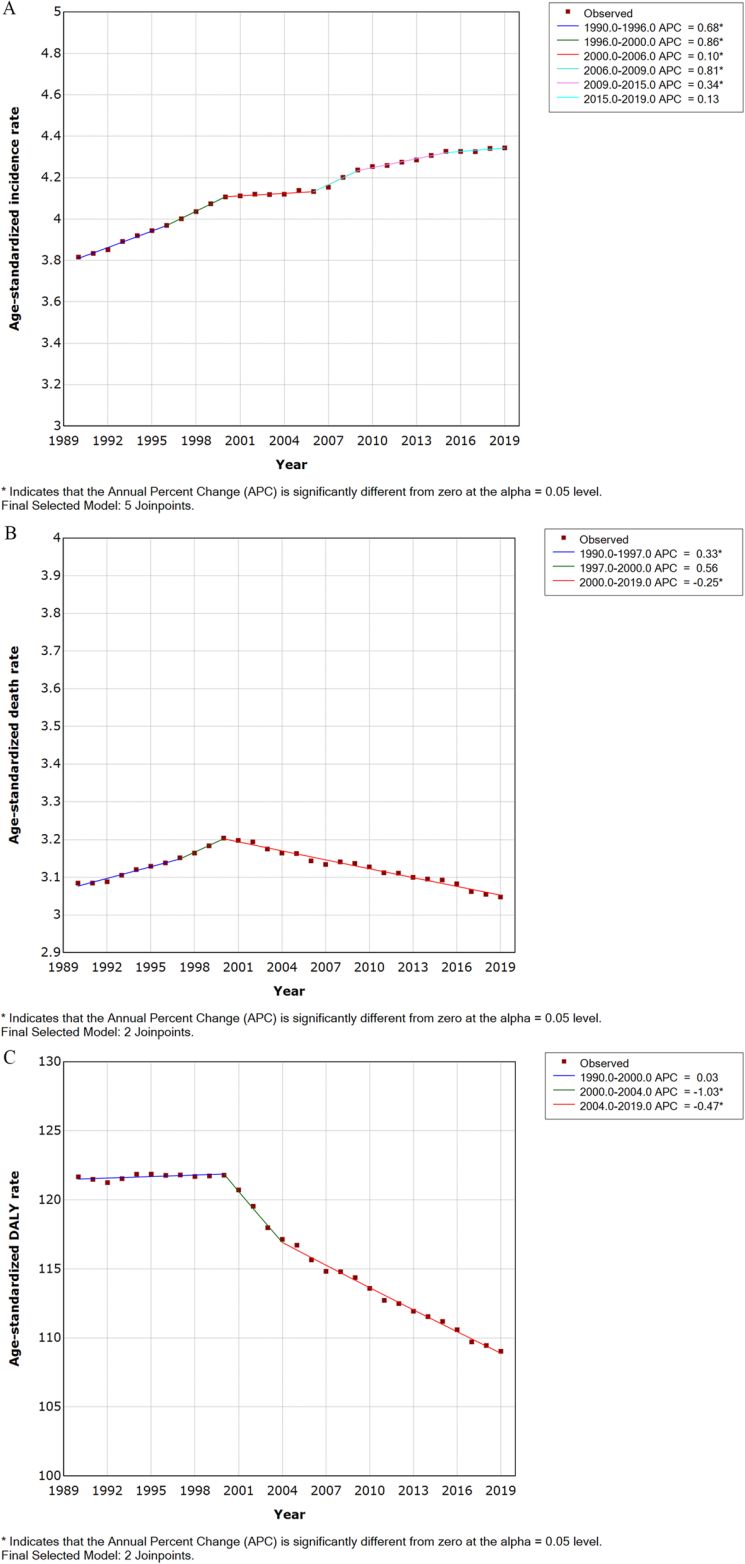
Trends for the ASIR (**A**), ASMR (**B**), and age-standardized DALY rate (**C**) of brain and central nervous system cancer worldwide from 1990 to 2019 calculated by Joinpoint regression analyses. Abbreviations: ASIR, age-standardized incidence rate; ASMR, age-standardized mortality rate; DALY, disability-adjusted life-year

Between 1990 and 2019, the ASIRs increased steadily in five SDI regions for both sexes (Table 2, Fig. 3). The ASMRs declined slightly in the high-and high-middle SDI regions and increased in the middle, low, and low-middle SDI regions (Table 2, Fig. 3). The age-standardized DALY rates declined significantly in the high and high-middle SDI regions and increased slightly in the low SDI regions (Table 2, Fig. 3). The results of the joinpoint regression analysis are displayed in Fig. 4. Globally, the ASIR increased during six periods (1990–1996: increased by an APC of 0.68%, Fig. 4A; 1996–2000: increased by 0.86%; 2000–2006: by 0.10%; 2006–2009: by 0.81%; 2009–2015: by 0.34%; and 2015–2019: by 0.13%). The ASMR first increased during two periods and then declined during one period (1990–1997: increased by 0.33%, Fig. 4B; 1997–2000: increased by 0.56%; and 2000–2019: declined by 0.25%). Similarly, the trend of increasing first and then decreasing was found in the age-standardized DALY rates (1990–2000: increased by 0.03%, Fig. 4C; 2000–2004: declined by 1.03%; and 2004–2019: declined by 0.47%). Overall, the APC values for these three indicators were small.

### Age and sex patterns

**Fig. 5:**
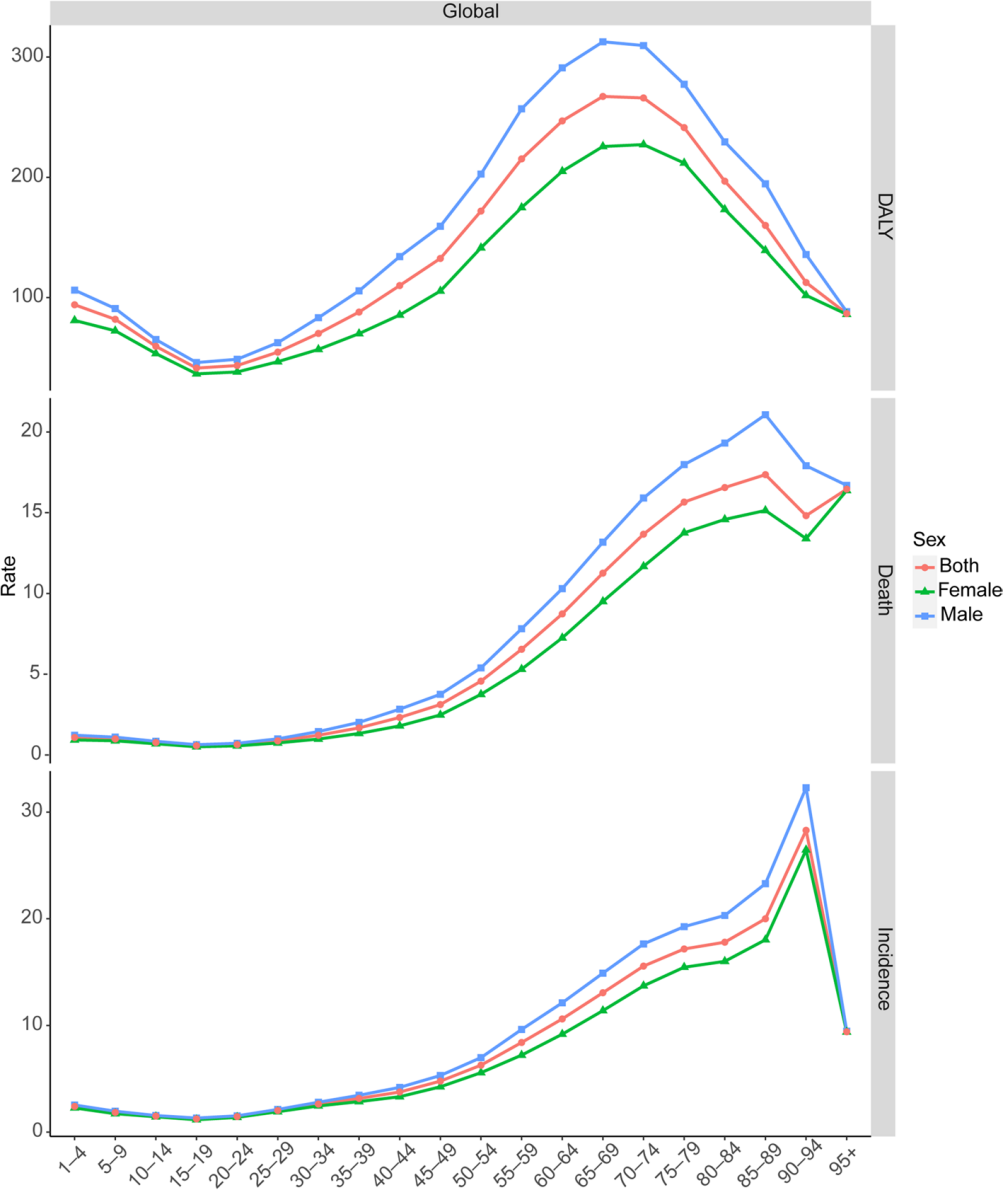
Age-standardized rates of brain and central nervous system cancer by sex and age in 2019 worldwide

Of the 347,992 global incident cases of brain and CNS cancers, 46.12% were female and 53.88% were male (death cases: 43.71% vs. 56.29%; DALYs: 42.11% vs. 57.89%). The number and age-standardized rates of brain and CNS cancers by age groups in 2019 are provided in Table 4. With regard to the age groups and gender, differences were observed between 1990–2019. Between 1990–2019, males showed higher age-standardized rates of incidence, mortality, and DALY than women across all the age groups at the global level (Fig. 3). Among the men, the highest ASIR was observed for those aged between 90 and 94 years (32.28 [18.85–39.18]), followed by the age group 85–89 (23.29 [14.06–27.94]), and the age group 80–84 (20.31 [12.37–24.05]) (Fig. 5). Among the women, the 90–94 age group showed the highest ASIR (26.45 [13.89–34.67]), followed by the 85–89 age group (18.03[9.89–23.04]), and the 80–84 age group (16.00 [9.06–19.74]) (Fig. 5). Among the men, the highest ASMR was observed for those aged 85–89 years (21.06 [13.03–24.71]), followed by those aged 80–84 years (19.31 [12.33–22.44]), and those aged 75–79 years (17.98 [11.92–20.58]) (Fig. 5). Among the women, people aged 95 years and older showed the highest ASMR (16.37 [9.16–20.73]), followed by those of the age group 85–89 years (15.14 [8.69–18.69]), and the age group 80–84 years (14.58[8.37–17.78]) (Fig. 5). Among the men, the highest age-standardized DALY rate was observed for the age group 65–69 years (312.69 [218.10–358.85]), followed by those of the age group 70–74 years (309.57 [212.27–353.01]), and those of the age group 60–64 years (291.02 [212.79–333.79]) (Fig. 5). Among the women, those of the age group 70–74 years (227.21 [147.32–259.84]) showed the highest age-standardized DALY rate, followed by those of the age group 65–69 years (225.62 [156.97–256.12]), and those of the age group 75–79 years (211.89 [129.65–247.50]) (Fig. 5). Figure ​5 also shows the age-standardized rates of incidence, mortality and DALY in 2019. Children and adolescents (0–19 years old) had the lowest ASIR and ASMR in both sexes. The ASIR increased with age (20–94 years), reaching the highest rate at approximately 94 years, and then declined slightly in the oldest age group (≥ 95 years). The ASMR increased with age (20–89 years), reaching the highest rate at approximately 89 years, and then declined slightly after 90 years of age. The ASIR and ASMR increased slowly with age in young adults (20–39 years old). After 40–89 years of age, the rates increased sharply in both sexes.

**Table 4:** The numbers and age-standardized rates of brain and central nervous system cancer by age groups in global in 2019

**Age-group (years)**	**Incidence numbers (95% UI)**	**Age-standardized incidence rate per 100, 000 (95% UI)**	**Death numbers (95% UI)**	**Age-standardized mortality rate per 100, 000(95% UI)**	**DALYs numbers (95% UI)**	**Age-standardized DALY rate per 100, 000 (95% UI)**
1–4	12,777 (9,721–15,697)	2.41 (1.83–2.96)	5,743 (4,409–7,173)	1.08 (0.83–1.35)	499,212 (382,409–624,039)	94.04 (72.04–117.55)
5–9	12,075 (9,432–14,009)	1.84 (1.44–2.14)	6,507 (5,073–7,612)	0.99 (0.77–1.16)	536,511 (418,519–627,993)	81.95 (63.92–95.92)
10–14	9,614 (7,588–11,043)	1.50 (1.18–1.72)	4,951 (3,928–5,701)	0.77 (0.61–0.89)	381,980 (303,177–439,587)	59.48 (47.21–68.45)
15–19	7,668 (5,892–8,722)	1.24 (0.95–1.41)	3,550 (2,754–4,038)	0.57 (0.44–0.65)	256,607 (199,225–291,767)	41.42 (32.16–47.09)
20–24	8,730 (6,667–9,876)	1.45 (1.11–1.65)	3,866 (3,001–4,332)	0.64 (0.50–0.72)	261,129 (202,952–291,748)	43.51 (33.82–48.61)
25–29	12,226 (9,622–13,785)	2.02 (1.59–2.28)	5,280 (4,117–5,874)	0.87 (0.68–0.97)	330,897 (257,685–368,650)	54.65 (42.56–60.89)
30–34	15,807 (12,575–17,735)	2.63 (2.09–2.95)	7,337 (5,794–8,151)	1.22 (0.96–1.35)	422,390 (332,721–468,223)	70.20 (55.29–77.81)
35–39	17,080 (13,415–19,275)	3.16 (2.48–3.56)	9,073 (7,089–10,161)	1.68 (1.31–1.88)	475,883 (372,257–532,417)	87.97 (68.81–98.42)
40–44	18,540 (14,426–21,063)	3.76 (2.92–4.27)	11,457 (8,887–12,802)	2.32 (1.80–2.59)	542,880 (421,238–607,202)	110.02 (85.37–123.05)
45–49	22,637 (17,911–25,646)	4.78 (3.78–5.41)	14,784 (11,590–16,442)	3.12 (2.45–3.47)	627,998 (493,075–699,085)	132.54 (104.07–147.55)
50–54	27,399 (21,754–31,029)	6.27 (4.98–7.10)	19,959 (15,711–22,196)	4.57 (3.60–5.08)	751,039 (591,047–834,173)	171.93 (135.31–190.97)
55–59	31,167 (24,357–34,925)	8.40 (6.56–9.41)	24,281 (18,796–26,780)	6.54 (5.07–7.22)	798,866 (618,020–881,664)	215.32 (166.58–237.64)
60–64	33,161 (25,104–37,321)	10.61 (8.03–11.94)	27,298 (20,523–30,061)	8.73 (6.57–9.62)	771,609 (579,571–849,181)	246.89 (185.44–271.71)
65–69	33,783 (24,655–37,861)	13.06 (9.53–14.64)	29,111 (21,433–32,153)	11.26 (8.29–12.43)	691,058 (509,668–763,069)	267.25 (197.10–295.10)
70–74	29,110 (20,435–33,069)	15.56 (10.92–17.68)	25,569 (18,244–28,531)	13.67 (9.75–15.25)	497,636 (353,795–555,238)	265.99 (189.11–296.78)
75–79	21,812 (14,356–25,136)	17.17 (11.30–19.78)	19,893 (13,492–22,540)	15.66 (10.62–17.74)	306,655 (207,165–347,636)	241.36 (163.05–273.61)
80–84	15,028 (9,172–17,961)	17.80 (10.86–21.28)	13,979 (8,782–16,319)	16.56 (10.40–19.33)	166,114 (104,353–194,117)	196.77 (123.61–229.94)
85–89	8,695 (5,085–10,676)	20.00 (11.69–24.55)	7,547 (4,596–9,011)	17.36 (10.57–20.72)	69,565 (42,202–82,986)	159.99 (97.06–190.86)
90–94	4,769 (2,716–6,042)	28.29 (16.11–35.84)	2,498 (1,463–3,038)	14.82 (8.68–18.02)	18,976 (10,986–23,237)	112.57 (65.17–137.84)
95 +	449 (260–563)	9.41 (5.44–11.80)	786 (455–977)	16.46 (9.53–20.46)	4,138 (2,406–5,141)	86.69 (50.41–107.70)
5–14	21,689 (17,066–24,894)	1.67 (1.32–1.92)	11,458 (9,085–13,291)	0.88 (0.70–1.02)	918,491 (729,458–1,064,866)	70.82 (56.25–82.11)
15–39	61,511 (48,214–69,132)	2.07 (1.62–2.33)	29,105 (22,982–32,317)	0.98 (0.77–1.09)	1,746,906 (1,382,424–1,938,407)	58.86 (46.58–65.31)
15–49	102,687 (80,890–115,094)	2.61 (2.06–2.92)	55,346 (43,754–61,463)	1.41 (1.11–1.56)	2,917,784 (2,305,066–3,247,571)	74.15 (58.58–82.53)
60–89	141,588 (100,138–160,569)	13.97 (9.88–15.85)	123,396 (88,809–137,612)	12.18 (8.77–13.58)	2,502,636 (1,826,935–2,767,736)	247.01 (180.32–273.18)

*Abbreviations*: *UI* Uncertainty interval, *DALY* Disability-adjusted life-year

### The relationship between the ASIR, ASMR, age-standardized DALY rate, and the SDI

**Fig. 6:**
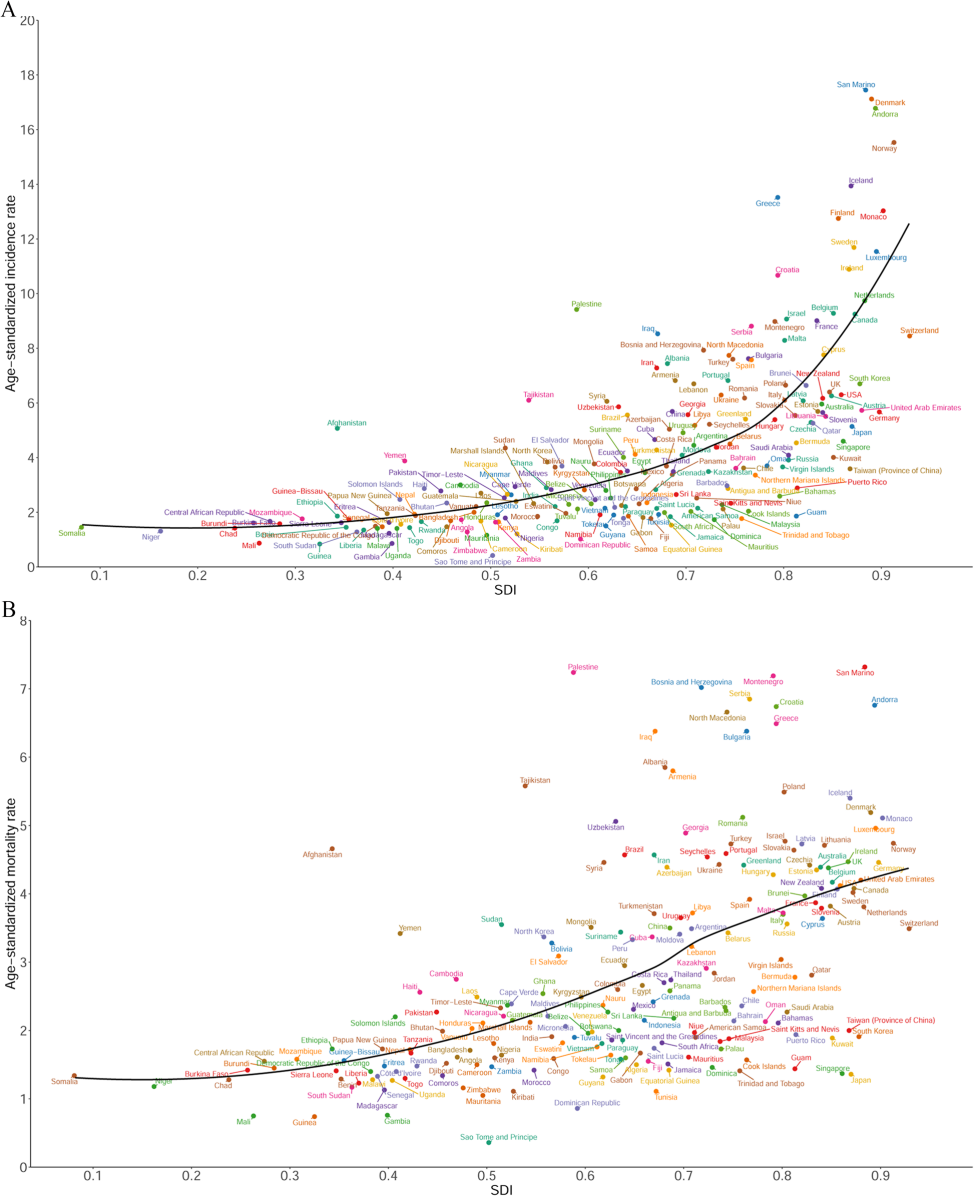
Age-standardized incidence rates (**A**) and age-standardized mortality rates (**B**) of brain and central nervous system cancer in 204 countries and territories in 2019 by SDI. Abbreviations: SDI, socio-demographic index

In 2019, there was a positive relationship between the ASIR and ASMR for brain and CNS cancers and the SDI of a country at the national level (Fig. 6A and B). Similar patterns were observed for the age-standardized DALY rate in relation to the SDI (Figure S1).

## Discussion

This study presented the latest incidence, mortality, disability, and trends for brain and CNS cancers at the global, regional, and country levels. The global incidence, deaths, and DALYs of brain and CNS cancers were shown to have increased significantly from 1990 to 2019. The global ASIR kept rising steadily, while the ASMR and age-standardized DALY rates declined.

Huge difference existed in all these estimates across the different regions and countries. The incident cases occurred mainly in the high, high-middle, and middle SDI regions or in the high-income and upper-middle-income regions, and especially in Western Europe (for example, in Iceland, Norway, Sweden, and Denmark). The highest incidences of brain and CNS cancers were observed in Western Europe (for example, in Iceland, Norway, Sweden, and Denmark) and the lowest incidences in Africa (for example, in Gambia, Guinea, and Sao Tome and Principe). These findings were consistent with those of previous studies [23]. The highest incidence rate of brain and CNS cancer in Western Europe can be explained by the higher socioeconomic status, higher medical levels, and higher quality of the tumor registration systems in these regions, which may have increased the detection rate of the brain tumors [24–26]. The medical level included the advancement of the hospital's diagnostic technology (MRI and CT scans) and the professional knowledge of the doctors [26]. Moreover, it is still noteworthy that the lack of timely and accurate diagnosis of brain and CNS cancer in those low-income regions (for example, in Gambia, Guinea, and Sao Tome and Principe) could explain the lowest incidence rate and thus lead to more patients who did not receive timely treatment, which means higher mortality for patients. Therefore, the variation of cancer burden due to region/country should be thought out in healthcare planning and resource allocation. Low-income regions or countries need more medical resources and access to medicine for better global health for brain and CNS cancer.

Although the results have been inconsistent, several studies have investigated the relationship between the incidence of brain cancers and socioeconomic status [11, 27, 28]. In this study, we found that the burden of brain and CNS cancers increased as the SDI (reflecting socioeconomic status) increased. High incidence, mortality, and DALY rates showed strong positive associations with the national per capita income levels, which probably related to better diagnostic facilities and access to treatment.

Males showed higher age-standardized rates of incidence, mortality, and DALY than females across the age groups, which was consistent with the results of previous studies [29, 30]. Such differences may have been due to genetic differences, lifestyle differences, and differences in medical attitudes and habits. We found that children and adolescents showed the lowest ASIR and ASMR for both sexes. The ASIR and ASMR increased with age, reaching the highest rate, followed by a slight decline in the oldest age group. Previous studies have reported that the incidence of anaplastic astrocytoma and glioblastoma increased with age, peaking in the 75–84 year age group [2, 8]. Older persons were also found to be less likely to be diagnosed with CNS cancer using microscopy, which may have affected the incidence rates [31].

There were some deficiencies in our study. First, epidemiological data on the histological types of brain and CNS cancers were lacked. There is a large heterogeneity in brain and CNS cancers (in terms of histology, grade, and clinical outcomes). However, we could not identify the different cancer burdens of the different histological types because of lacking relevant detailed information. Second, data on risk factors (such as environmental and occupational exposure factors) associated with brain and CNS cancers were not available in the database, and we could not explore the risk factors that contributed to the deaths and DALYs of the brain and CNS cancers in this study.

## Conclusions

In conclusion, brain and CNS cancers remain a major public health burden, particularly in high-income regions. The global incidence, deaths, and DALYs of brain and CNS cancers were shown to have increased significantly from 1990 to 2019. The global ASIR kept rising steadily, while the ASMR and age-standardized DALY rate declined over the past three decades.

## Supplementary Information

**Additional file 1: Figure S1.** Age-standardized DALY rates of brain and central nervous system cancer in 204 countries and territories in 2019 by SDI. Abbreviations: DALY, disability-adjusted life-year; SDI, socio-demographic index.

## Data Availability

The datasets supporting the conclusions of this article are available in the http://ghdx.healthdata.org/gbd-results-tool.

## Abbreviations

CNS: Central nervous system
DALY: Disability-adjusted life-year
GBD: Global Burden of Disease
ASIR: Age-standardized incidence rate
ASMR: Age-standardized mortality rate
SDI: Sociodemographic index
APC: The annual percent change
