# Supplementary figures and images for: Burden and trends of brain and central nervous system cancer from 1990 to 2019 at the global, regional, and country levels
Source: Arch Public Health. 2022 Sep 17;80:209. doi: 10.1186/s13690-022-00965-5 (PMC9482735; doi:10.1186/s13690-022-00965-5)

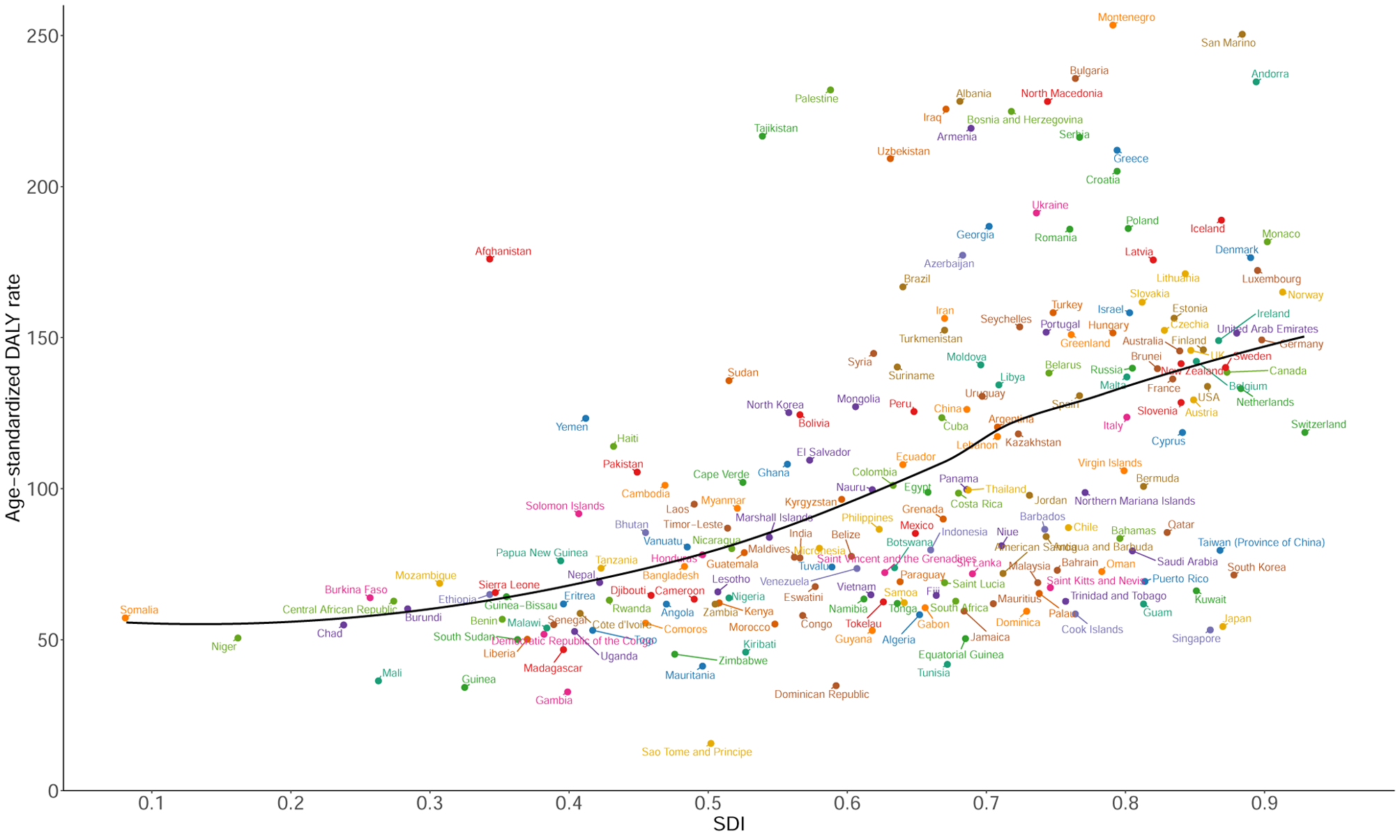

Supplement: Supplementary file 1 — Additional file 1: Figure S1. Age-standardized DALY rates of brain and central nervous system cancer in 204 countries and territories in 2019 by SDI. Abbreviations: DALY, disability-adjusted life-year; SDI, socio-demographic index. [file 13690_2022_965_MOESM1_ESM.tif]
